# Suppression of neuropathic pain in the circadian clock–deficient *Per2^m/m^* mice involves up-regulation of endocannabinoid system

**DOI:** 10.1093/pnasnexus/pgad482

**Published:** 2024-01-17

**Authors:** Wakaba Yamakawa, Sai Yasukochi, Yuya Tsurudome, Naoki Kusunose, Yuta Yamaguchi, Akito Tsuruta, Naoya Matsunaga, Kentaro Ushijima, Satoru Koyanagi, Shigehiro Ohdo

**Affiliations:** Department of Pharmaceutics, Faculty of Pharmaceutical Sciences, Kyushu University, Fukuoka, 812-8582, Japan; Department of Pharmaceutics, Faculty of Pharmaceutical Sciences, Kyushu University, Fukuoka, 812-8582, Japan; Division of Pharmaceutics, Faculty of Pharmaceutical Sciences, Sanyo-Onoda City University, Yamaguchi, 756-0884, Japan; Department of Pharmaceutics, Faculty of Pharmaceutical Sciences, Kyushu University, Fukuoka, 812-8582, Japan; Department of Pharmaceutics, Faculty of Pharmaceutical Sciences, Kyushu University, Fukuoka, 812-8582, Japan; Department of Pharmaceutics, Faculty of Pharmaceutical Sciences, Kyushu University, Fukuoka, 812-8582, Japan; Department of Glocal Healthcare Science, Faculty of Pharmaceutical Sciences, Kyushu University, Fukuoka, 812-8582, Japan; Department of Clinical Pharmacokinetics, Faculty of Pharmaceutical Sciences, Kyushu University, Fukuoka, 812-8582, Japan; Division of Pharmaceutics, Faculty of Pharmaceutical Sciences, Sanyo-Onoda City University, Yamaguchi, 756-0884, Japan; Department of Pharmaceutics, Faculty of Pharmaceutical Sciences, Kyushu University, Fukuoka, 812-8582, Japan; Department of Glocal Healthcare Science, Faculty of Pharmaceutical Sciences, Kyushu University, Fukuoka, 812-8582, Japan; Department of Pharmaceutics, Faculty of Pharmaceutical Sciences, Kyushu University, Fukuoka, 812-8582, Japan

**Keywords:** circadian clock, clock gene, Period2, neuropathic pain, α1D-adrenergic receptor, 2-arachidonoylglycerol, endocannabinoid system

## Abstract

Neuropathic pain often results from injuries and diseases that affect the somatosensory system. Disruption of the circadian clock has been implicated in the exacerbation of the neuropathic pain state. However, in this study, we report that mice deficient in a core clock component *Period2* (*Per2^m/m^* mice) fail to develop tactile pain hypersensitivity even following peripheral nerve injury. Similar to male wild-type mice, partial sciatic nerve ligation (PSL)-*Per2^m/m^* male mice showed activation of glial cells in the dorsal horn of the spinal cord and increased expression of pain-related genes. Interestingly, α1D-adrenergic receptor (α1D-AR) expression was up-regulated in the spinal cord of *Per2^m/m^* mice, leading to increased production of 2-arachidonoylglycerol (2-AG), an endocannabinoid receptor ligand. This increase in 2-AG suppressed the PSL-induced tactile pain hypersensitivity. Furthermore, intraspinal dorsal horn injection of adeno-associated viral vectors expressing α1D-AR also attenuated pain hypersensitivity in PSL-wild-type male mice by increasing 2-AG production. Our findings reveal an uncovered role of the circadian clock in neuropathic pain disorders and suggest a link between α1D-AR signaling and the endocannabinoid system.

**Significance Statement**

Neuropathic pain, often associated with problems in the somatosensory system, is thought to be exacerbated by disruption of the circadian clock. However, unexpectedly, mice deficient in core circadian clock component *Period2* (*Per2^m/m^*) did not produce tactile pain hypersensitivity after peripheral nerve injury. Despite similarities in spinal glial cell activation and pain-related gene expression with wild-type mice, *Per2^m/m^* exhibited elevated α1D-adrenergic receptor (α1D-AR) expression in the spinal cord, leading to increased production of 2-arachidonoylglycerol, an endocannabinoid receptor ligand, which suppressed nerve injury-induced pain hypersensitivity. The present findings reveal a previously unknown role of the circadian clock in neuropathic pain disorders and also suggest a molecular link between α1D-AR signaling and the endocannabinoid system.

## Introduction

Circadian clock machinery is present in most organisms and provides an adaptive mechanism to coordinate their physiological and behavioral functions with predictable changes in the environment (1). In mammals, the circadian rhythms in physiological functions are governed by a molecular oscillator driven by a transcriptional–translational feedback loop consisting of clock genes (2). The gene products of *Clock* and *Arntl* (also known as *Bmal1*) form a heterodimer and promote the transcription of *Period* (*Per*) and *Cryptochrome* (*Cry*) genes. When PER and CRY proteins reach critical levels, they repress CLOCK/BMAL1-mediated transactivation. Since the expression of up to 15% of genes is under the control of the molecular circadian clock, it is not surprising that clock gene dysfunction affects the onset and/or state of various diseases (2, 3). In fact, disruption of circadian rhythms has been shown to negatively affect health and increase the risk of developing cancer (4), cardiovascular disease (5), and diabetes (6).

Pain is a warning sign of tissue damage in the body, but when it persists after the body has healed or for an unknown reason, it is referred to as chronic pain (7). The number of patients with chronic pain disorders is increasing worldwide, and ∼25% of the world’s population currently suffers from chronic pain (8, 9). Neuropathic pain is one of the most debilitating chronic pain conditions caused by trauma (10), diabetes (11), cancer cell infiltration into the nerve (12), and herpes virus infections (13). A troublesome hallmark symptom of neuropathic pain is hypersensitivity to normally innocuous stimuli, which is known as “tactile allodynia” (14, 15) and often refractory to common analgesic therapies (16). Diurnal variation in pain sensitivity has also been observed in patients with cancer (17), rheumatoid arthritis (18), diabetic neuropathy (19), fibromyalgia (20), and multiple sclerosis (21). Therefore, dysfunction of the circadian clock is implicated in the exacerbation of maladaptive pain (22, 23). However, our understanding of the role of clock genes in the development of neuropathic pain is limited.

Mice with a mutated *Per2* gene (*Per2^m/m^*) exhibit an abnormal rhythm in physiology and behavior (24, 25). Unexpectedly, we found that male *Per2^m/m^* mice failed to develop tactile allodynia even after partial sciatic nerve ligation (PSL). As observed in male wild-type mice, sciatic nerve ligation also induced the activation of glial cells and expression of pain-related genes in the spinal cord of male *Per2^m/m^* mice, but they also exhibited an increase in α1D-adrenergic receptor (α1D-AR) expression and 2-arachidonoylglycerol (2-AG) levels in the spinal cord, which prevented the PSL-induced tactile pain hypersensitivity. Collectively, our present findings suggest a molecular link connecting the circadian clock to the endogenous analgesic system and provide effective approaches for the treatment of neuropathic pain.

## Results

### *Per2^m/m^* mice fail to produce neuropathic tactile allodynia

**Fig. 1.**
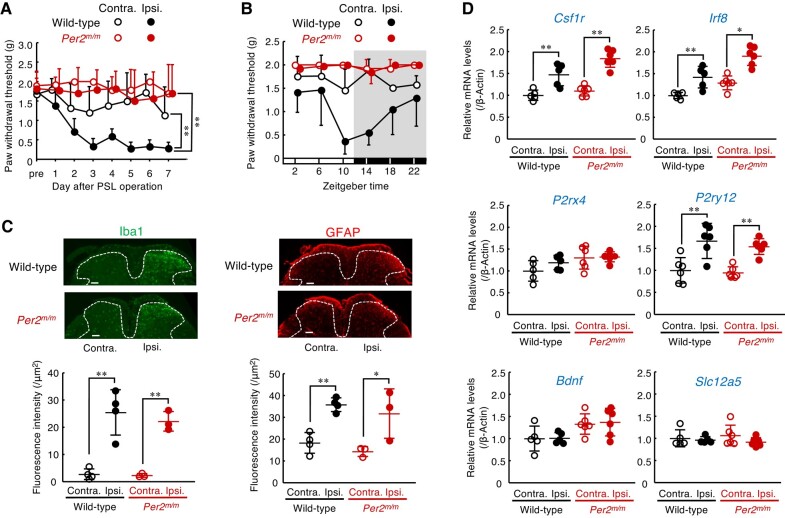
*Per2^m/m^* mice fail to produce neuropathic pain hypersensitivity. A) The withdrawal threshold of contralateral (contra.) and ipsilateral (ispi.) hind paws of male wild-type and male *Per2^m/m^* mice following PSL. The threshold was assessed at ZT10. Values are shown as means with SD (*n* = 6). **P < 0.01, significant difference between the two groups (group: *F*_3,158_ = 53.581; *P* < 0.001, day; *F*_7,158_ = 4.166; *P* < 0.001, interaction; *F*_21,158_ = 1.919; *P* = 0.013, two-way ANOVA with Tukey–Kramer's post hoc test). B) Temporal profiles of the PWT in male wild-type and male *Per2^m/m^* mice. The withdrawal threshold of contralateral (contra.) and ipsilateral (ispi.) hind paws of wild-type and *Per2^m/m^* mice were assessed on day 7 after PSL. Values are shown as means with SD (*n* = 5–6). Significant time-dependent variation is observed in the withdrawal threshold of the ipsilateral hind paw of wild-type but not *Per2^m/m^* mice (*F*_5,24_ = 4.137; *P* = 0.008 for wild-type, *F*_5,30_ = 1.451; *P* = 0.235 for *Per2^m/m^*, two-way ANOVA). C) Microglia and astrocyte activation in the spinal dorsal horn of male wild-type and male *Per2^m/m^* mice. Immunofluorescence staining with Iba1; a marker of microglia, and glial fibrillary acidic protein (GFAP); a marker of astrocyte, in the spinal cord at ZT10. Scale bar, 100 µm. The dorsal horn areas are surrounded by the dashed line. The lower panel shows the fluorescent intensity of Iba1-positive and GFAP-positive cells. Values are shown as mean with SD (*n* = 3–4). **P < 0.01, *P < 0.05, a significant difference between the two groups (*F*_3,10_ = 22.099, *P* < 0.001 for Iba1, *F*_3,10_ = 10.308, *P* = 0.002 for GFAP, one-way ANOVA with Tukey–Kramer's post hoc test). D) The mRNA levels of *Csf1r*, *Irf8*, *P2rx4*, *P2ry12*, *Bdnf*, and *Slc12a5* in the spinal cord of male wild-type and male *Per2^m/m^* mice. The mRNA levels were assessed at ZT10. Values are shown with SD (*n* = 5–6). **P < 0.01, *P < 0.05, significant difference between two groups (*F*_3,18_ = 25.413, *P* < 0.001 for *Csf1r*, *F*_3,18_ = 22.682, *P* < 0.001 for *Irf8*, *F*_3,18_ = 2.877, *P* = 0.065 for *P2rx4*, *F*_3,20_ = 18.682, *P* < 0.001 for *P2ry12*, *F*_3,18_ = 3.466, *P* = 0.038 for *Bdnf*, *F*_3,18_ = 0.871, *P* = 0.475 for *Slc12a5*; one-way ANOVA with Tukey–Kramer's post hoc test).

To determine the pathological relevance of the clock gene in neuropathic pain hypersensitivity, we used *Per2^m/m^* mice and assessed the severity of peripheral nerve injury-induced tactile allodynia. Both male wild-type and male *Per2^m/m^* mice underwent PSL in the right hindlimb, a well-established peripheral nerve injury model that produces tactile allodynia (26). The background of these mice was the same as that of the mice used in the previous report demonstrating diurnal exacerbation of neuropathic tactile allodynia (27). All animals were maintained on a 12-h light–dark cycle (ZT, zeitgeber time; ZT0, lights on; ZT12, lights off). Following PSL, the withdrawal threshold to tactile stimuli in the ipsilateral hind paw was assessed daily at ZT10, when neuropathic pain hypersensitivity is typically exacerbated in rodents (27). We also attempted to investigate female mice, but the mean value of the paw withdrawal threshold (PWT) of *Per2^m/m^* female mice following PSL exhibited a large individual variation. Thus, in this study, we used male mice to evaluate the role of the *Per2* gene in the pathological states of neuropathic pain hypersensitivity. The withdrawal threshold of the ipsilateral hind paw, but not that of the contralateral hind paw, of wild-type mice was significantly decreased after PSL, revealing the production of tactile allodynia (*P* < 0.01, Fig. 1A). In contrast, the withdrawal threshold of the ipsilateral hind paw of *Per2^m/m^* mice did not decrease after PSL. No significant reduction in the withdrawal threshold of the ipsilateral hind paw of *Per2^m/m^* mice was observed not only at ZT10 (Fig. 1A) but also at other examined time points (Fig. 1B). These results indicate that *Per2* is involved in the mechanism producing neuropathic pain hypersensitivity rather than its diurnal regulation. Importantly, before PSL, *Per2^m/m^* mice showed normal mechanical sensitivity comparable to wild-type mice (Fig. 1A). Furthermore, hot plate and capsaicin tests revealed that acute physiological pain responses were not different between male wild-type and male *Per2^m/m^* mice (Fig. S1A), suggesting that *Per2* gene deficiency does not cause a defect in general pain sensation.

In response to PSL, spinal glial cells transformed into a reactive state through a sequence of cellular and molecular changes. These changes include morphological hypertrophy, proliferation, and alteration in gene expression (28, 29). On day 7 after PSL, significant increases in the number of Iba1-positive cells, a marker of microglia, and glial fibrillary acidic protein (GFAP)-positive cells, a marker of astrocytes, were observed in the ipsilateral side of the spinal cord in male wild-type mice (*P* < 0.01, *P* < 0.05, each; Fig. 1C). We also detected elevations in the mRNA levels of colony-stimulating factor 1 receptor (*Csf1r*; *P* < 0.01), interferon regulatory factor 8 (*Irf8*; *P* < 0.01), and metabotropic P2Y12 receptor (*P2ry12*; *P* < 0.01) in the ipsilateral side of the spinal cord in male wild-type mice (Fig. 1D). Although male *Per2^m/m^* mice failed to produce tactile allodynia, *Per2* gene deficiency had a negligible effect on the PSL-induced increase in the number of glial cells (Fig. 1C), as well as the expression of neuropathic pain-related genes in the ipsilateral side of the spinal cord (Fig. 1D). These results indicate that the deficiency in *Per2* gene prevents the production of neuropathic pain hypersensitivity without affecting morphological or molecular alterations in spinal glial cells.

### Enhanced descending noradrenergic pain suppression pathways in *Per2^m/m^* mice

**Fig. 2.**
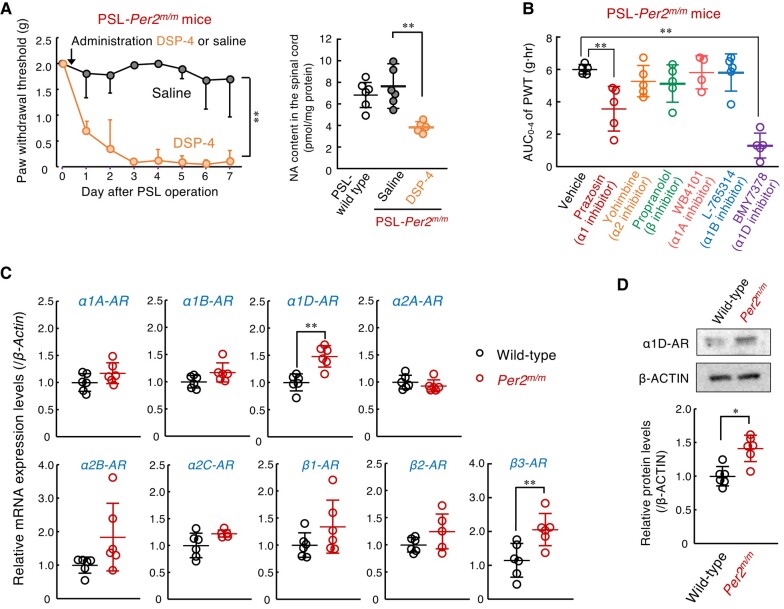
Enhancement of descending noradrenergic pain suppression pathways in *Per2^m/m^* mice. A) Production of tactile allodynia and spinal noradrenaline (NA) content of PSL-*Per2^m/m^* male mice after intraperitoneal injection of DSP-4. One day after the PSL operation, DSP-4 (50 mg/kg) was intraperitoneally administrated to PSL-*Per2^m/m^* mice. The left panel shows the withdrawal threshold of the ipsilateral hind paw of DSP-4 administrated PSL-*Per2^m/m^* at ZT10. Values are shown as mean with SD (*n* = 6). **P < 0.01, a significant difference between the two groups (drug; *F*_1,79_ = 484.229; *P* < 0.001, days; *F*_7,79_ = 14.844; *P* < 0.001, interaction; *F*_7,79_ = 10.704; *P* < 0.001, two-way ANOVA). The left panel shows NA content in the spinal cord of PSL-wild-type male mice and DSP-4 administrated PSL-*Per2^m/m^* male mice. The NA content was assessed at ZT10 on day 7 after DSP-4 administration. Values are shown as mean with SD (*n* = 5–6). **P < 0.01, a significant difference between the two groups (*F*_2,14_ = 8.127, *P* = 0.005, one-way ANOVA with Tukey–Kramer's post hoc test). B) Effects of selective adrenaline receptor antagonists on the withdrawal threshold of ipsilateral hind paws of PSL-*Per2^m/m^* male mice. The PWT of ipsilateral side of PSL-*Per2^m/m^* mice was assessed up to 4 h after intrathecal injection of each antagonist at ZT10. The dosage of each drug was determined based on previous reports as described in materials and methods section. The AUC of PWT after the drug injection was calculated using the trapezoidal rule. Values are shown as means with SD (*n* = 4–5). **P < 0.01, a significant difference from vehicle-treated group (*F*_6,27_ = 13.985, *P* < 0.001, ANOVA with Dunnett post hoc test). C) The mRNA levels of adrenaline receptors (*α1A*, *α1B*, *α1D*, *α2A*, *α2B*, *α2C*, *β1*, *β2*, *β3*) in the spinal cord of male wild-type and male *Per2^m/m^* mice at ZT10. **P < 0.01, a significant difference between the two groups (Student's t test). D) The protein levels of α1D-AR in the spinal cord of male wild-type and male *Per2^m/m^* mice at ZT10. The expression levels of α1D-AR protein were normalized to that of β-ACTIN. *P < 0.05, a significant difference between the two groups (Student's t test).

Descending noradrenergic inhibitory pathways from the locus coeruleus to the dorsal horn of the spinal cord function as an endogenous analgesic system (30). Treatment with *N*-(2-chloroethyl)-*N*-ethyl-2-bromobenzylamine (DSP-4), a noradrenergic neurotoxin, produced tactile allodynia in PSL-*Per2^m/m^* male mice, accompanied by a reduction of noradrenaline contents in the spinal cord (Fig. 2A), while having a negligible effect on serotonin levels (Fig. S2). Since spinal noradrenaline levels in *Per2^m/m^* mice were comparable with those in wild-type mice, we raised the possibility that enhancement of noradrenergic signaling by activation of a specific type of adrenergic receptor (AR) prevents the PSL-induced tactile pain hypersensitivity in *Per2^m/m^* mice. To investigate this possibility, male *Per2^m/m^* mice were injected intrathecally with each type of AR antagonist and the area under the curve (AUC) of the withdrawal threshold of the ipsilateral hind paw was assessed up to 4 h after drug injection. Intrathecal injection (i.th.) of the α1-AR antagonist prazosin (30 nmol/mouse), but neither the α2-AR antagonist yohimbine (10 nmol/mouse, i.th.) nor the β-AR antagonist propranolol (100 nmol/mouse, i.th.) significantly decreased the PWT of PSL-*Per2^m/m^* mice (*P* < 0.01, Fig. 2B), suggesting that α1-AR is involved in the mechanism underlying the failure of PSL-induced tactile pain hypersensitivity in *Per2^m/m^* mice.

Because three α1-AR subtypes were expressed in the spinal cord of *Per2^m/m^* mice (Fig. 2C), we also examined the effects of each type of α1-AR antagonist on the withdrawal threshold of the ipsilateral hind paw of PSL-*Per2^m/m^* male mice. The PWT of PSL-*Per2^m/m^* mice was significantly decreased after intrathecal injection of the α1D-AR antagonist BMY7378 (0.1 nmol/mouse), but either the α1A-AR antagonist WB4101 (100 nmol/mouse, i.th.) or the α1B-AR antagonist L-765314 (0.25 nmol/mouse, i.th.) had a negligible effect on the PWT of PSL-*Per2^m/m^* mice (Fig. 2B). Spinal expression of α1D-AR was increased in *Per2^m/m^* mice (Fig. 2C and D), indicating that α1D-AR signaling in the spinal cord is enhanced and contributes to the prevention of PSL-induced tactile pain hypersensitivity in *Per2^m/m^* mice. Although a significant increase in the spinal expression of β3-AR was also observed in *Per2^m/m^* mice (Fig. 2C), the β-AR antagonist propranolol failed to produce tactile allodynia (Fig. 2B). Therefore, we further focused on the role of α1D-AR in the insensitivity of male *Per2^m/m^* mice to neuropathic pain.

### The α1D-AR-mediated production of 2-AG is involved in the insensitivity of *Per2^m/m^* mice to neuropathic pain

**Fig. 3.**
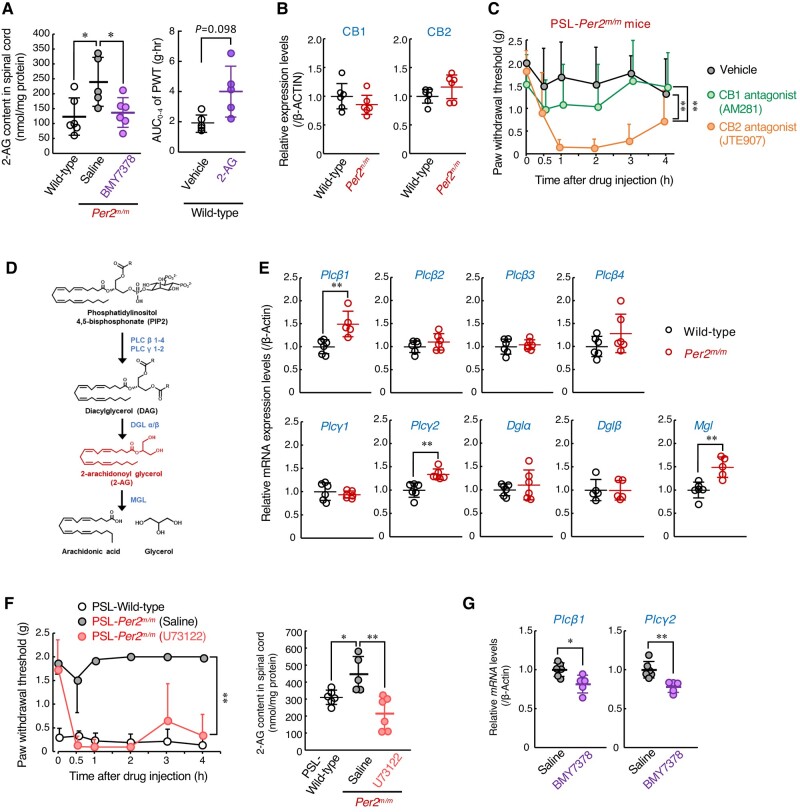
α1D-AR-mediated production of 2-AG is involved in the insensitivity of *Per2^m/m^* mice with neuropathic pain. A) Left panel shows the 2-AG content in the spinal cord of male wild-type mice and α1D-AR antagonist BMY7378 (0.1 nmol/mouse, i.th.)-injected PSL-*Per2^m/m^* male mice. The 2-AG content was assessed at 2 h after drug injection at ZT10. Values are shown as mean with SD (*n* = 6). *P < 0.05, a significant difference between the two groups (*F*_2,15_ = 10.586, *P* = 0.001, one-way ANOVA with Tukey–Kramer's post hoc test). Right panel shows the effects of 2-AG on the withdrawal threshold of ipsilateral hind paws of PSL-wild-type male mice. The PWT of ipsilateral side of PSL-wild-type mice was assessed up to 4 h after intrathecal injection of 2-AG (50 µg/mouse) at ZT10. The AUC of PWT after the drug injection was calculated using the trapezoidal rule. Values are shown as means with SD (*n* = 5). The *P*-value was calculated using Student’s t test. B) The protein levels of cannabinoid receptor 1 (CB1) and CB2 in the spinal cord of male wild-type and male *Per2^m/m^* mice at ZT10. The expression levels of CB1 and CB2 proteins were normalized to that of β-ACTIN. The original images of western blot are presented in Fig. S5. Values are shown as mean with SD (*n* = 6). C) The withdrawal threshold of ipsilateral hind paw of PSL-*Per2^m/m^* male mice was assessed before and after intrathecal injection of CB1 antagonist, AM281 (20 nmol/mouse), and CB2 antagonist, JTE907 (20 nmol/mouse) at ZT 10. Values are shown as mean with SD (*n* = 5–6). **P < 0.01, a significant difference between the two groups (drug; *F*_2,84_ = 19.645; *P* < 0.001, time point; *F*_5,84_ = 3.783; *P* = 0.004, interaction; *F*_10,84_ = 1.701; *P* = 0.094, two-way ANOVA with Tukey–Kramer's post hoc test). D) Schematic illustration indicating the pathways of 2-AG synthesis and degradation. E) The mRNA levels of 2-AG synthase and degradation enzyme (*Plcβ1*, *Plcβ2*, *Plcβ3*, *Plcβ4*, *Plcγ1*, *Plcγ2*, *Dglα*, *Dglβ*, and *Mgl*) in the spinal cord of male wild-type and male *Per2^m/m^* mice at ZT 10. Values are shown as means with SD (*n* = 6). **P < 0.01, significant difference between the two groups (Student's t test). F) Left panel shows the withdrawal threshold of ipsilateral hind paw of PSL-wild-type male mice and PSL-*Per2^m/m^* male mice after intrathecally injected with PLC inhibitor U73122 (10 nmol/mouse) or saline at ZT10. Values are shown as mean with SD (*n* = 5–6). **P < 0.01, a significant difference between the two groups (drug; *F*_1,54_ = 206.345; *P* < 0.001, time point; *F*_5,54_ = 8.620; *P* < 0.001; interaction; *F*_5,54_ = 7.792; *P* < 0.001, two-way ANOVA). Right panel shows the 2-AG content in the spinal cord of PSL-wild-type male mice and U73122 (10 nmol/mouse, i.th.)-injected PSL-*Per2^m/m^* mice. The 2-AG content was assessed 2 h after drug injection at ZT10. Values are shown as mean with SD (*n* = 5–6). **P < 0.01, *P < 0.05, a significant difference between the two groups (*F*_2,14_ = 10.273; *P* = 0.002, one-way ANOVA with Tukey–Kramer's post hoc test). G) The mRNA levels of 2-AG synthase (*Plcβ1* and *Plcγ2*) in the spinal cord of male *Per2^m/m^* mice at 2 h after injection of vehicle or BMY7378 (0.1 nmol/mouse, i.th.) at ZT10. Values are shown as mean with SD (*n* = 6). **P < 0.01, *P < 0.05, a significant difference between the two groups (Student's t test).

α1-ARs are members of the Gq protein-coupled receptor (GPCR) superfamily that, upon stimulation, activate phospholipase C and generate second lipid mediators (31). To search for endogenous analgesic substances whose production is under the control of α1D-AR, we conducted mass spectrometry analysis for lipid mediators in the spinal cord of mice. Our analytical system detected 24 lipid mediators in both the male wild-type and male *Per2^m/m^* mice (Table S1). Among them, 2-AG was significantly abundant in the spinal cord of *Per2^m/m^* mice (*P* < 0.05). Intrathecal injection of the α1D-AR antagonist BMY7378 altered the spinal levels for 10 of the 24 substances (Table S1). While the levels of most substances were increased by the injection of BMY7378, only the 2-AG levels were significantly decreased (Fig. 3A, left), suggesting that the production of 2-AG in *Per2^m/m^* mice is up-regulated via α1D-AR signaling. Intrathecal injection of 2-AG (50 g/mouse) into PSL-wild-type mice prevented tactile allodynia (Fig. 3A, right), but the same amount of 2-AG injection was ineffective in suppressing heat- and capsaicin-induced pain (Fig. S1B). Therefore, we further investigated the role of 2-AG in the α1D-AR-mediated antiallodynic effect.

2-AG is an endogenous cannabinoid receptor ligand and is implicated as an analgesic substance (32, 33). The expression levels of cannabinoid receptor-1 (CB1) and CB2 in the spinal cord of male *Per2^m/m^* mice were comparable with those of male wild-type mice (Fig. 3B), but intrathecal injection of either the CB1 antagonist AM281 (20 nmol/mouse) or the CB2 antagonist JTE907 (20 nmol/mouse) significantly decreased the PWT of PSL-*Per2^m/m^* mice (*P* < 0.01, each; Fig. 3C). A more potent analgesic effect was detected after injection of the CB2 antagonist. These results suggest that the enhancement of 2-AG production in the spinal cord of male *Per2^m/m^* mice is also involved in the underlying mechanism of their insensitivity to neuropathic pain.

2-AG is synthesized from membrane phospholipids by sequential activation of phospholipase C (PLCβ1–4 and PLCγ1–2) and diacylglycerol lipase α/β (DGLα/β), whereas monoacylglycerol lipase (MGL) is critical for the degradation of 2-AG (Fig. 3D). The mRNA levels of *Plcβ1* and *Plcγ2* were significantly increased in the spinal cord of *Per2^m/m^* mice (*P* < 0.01, each; Fig. 3E). Although the mRNA levels of the 2-AG degradation enzyme MGL were also elevated in *Per2^m/m^* mice, intrathecal injection of PLC inhibitor U73122 (10 nmol/mouse) significantly produced pain hypersensitivity in PSL-*Per2^m/m^* male mice (*P* < 0.01, each; Fig. 3F, left), which was accompanied by a reduction of 2-AG levels in the spinal cord (*P* < 0.01, Fig. 3F, right). Moreover, the spinal expressions of *Plcβ1* and *Plcγ2* mRNA in *Per2^m/m^* mice were also decreased by injections of the α1D-AR antagonist (Fig. 3G), indicating that α1D-AR-mediated production of 2-AG in the spinal cord contributes to the prevention of PSL-induced tactile pain hypersensitivity in *Per2^m/m^* male mice.

### The α1D-AR-mediated production of 2-AG in spinal astrocytes is under the control of Per2

**Fig. 4.**
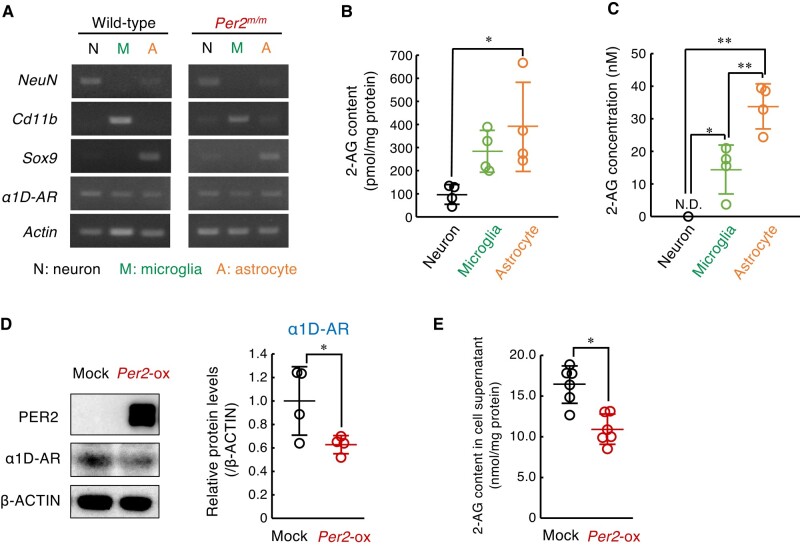
Per2 regulates the α1D-AR-mediated production of 2-AG in the spinal astrocytes. A) The expression of *α1D-AR* mRNA in neurons, microglia, and astrocytes of the spinal cord of male wild-type and male *Per2^m/m^* mice. Separation of each cell fraction was confirmed by the expression of marker genes *NeuN*; neuron marker, *CD11b*; microglia marker, and *Sox9*; astrocyte maker. B) The 2-AG in the neurons, microglia, and astrocytes of the spinal cord of male wild-type mice. Values are shown as mean with SD (*n* = 4). *P < 0.05, significant difference between the two groups (*F*_2,9_ = 5.578; *P* = 0.027, one-way ANOVA with Tukey–Kramer's post hoc test). C) The concentration of 2-AG in the culture media of neurons, microglia and astrocytes. Values are shown as mean with SD (*n* = 4). **P < 0.01, *P < 0.05, a significant difference between the two groups (*F*_2,9_ = 33.232, *P* < 0.001, ANOVA with Tukey–Kramer's post hoc test). D) The protein levels of α1D-AR in Per2-overexpressing (Per2-ox) astrocytes. Values are shown as means with SD (*n* = 4). *P < 0.05, a significant difference between the two groups (Student's t test). The original images of western blot are presented in Fig. S6. E) The concentration of 2-AG in the culture media of *Per2*-ox astrocytes. Values are shown as mean with SD (*n* = 6). *P < 0.05, a significant difference compared between the two groups (Student's t test).

To identify the cell types producing 2-AG in the spinal cord, we isolated astrocytes (CD11b^−^/ACSA-2^+^), microglia (CD45^Med^/CD11b^+^), and neurons from both male wild-type and male *Per2^m/m^* mice by combining magnetic-activated cell sorting and fluorescence activated cell sorting (Fig. S3). The expression of α1D-AR mRNA was detected in all isolated cell fractions (Fig. 4A). All isolated cells contained 2-AG, but the contents in astrocytic fraction were higher than those in the microglial and neuronal fractions (Fig. 4B). Similarly, higher levels of 2-AG were also observed in the media of cultured astrocytes (Fig. 4C). Therefore, we investigated whether α1D-AR expression and 2-AG production in astrocytes are altered by modulation of Per2 expression levels. Because western blotting with anti-mouse Per2 antibodies did not reveal the endogenous protein in cultured astrocytes, cells were infected with retrovirus expressing *Per2* gene to investigate the effect of Per2 protein on the expression of α1D-AR. The expression of Per2 protein was associated with significant decrease in the protein levels of α1D-AR (Fig. 4D) and 2-AG content in cultured astrocytes (Fig. 4E). This result was consistent with the *in vivo* findings that Per2 acts as a negative regulator of α1D-AR expression.

**Fig. 5.**
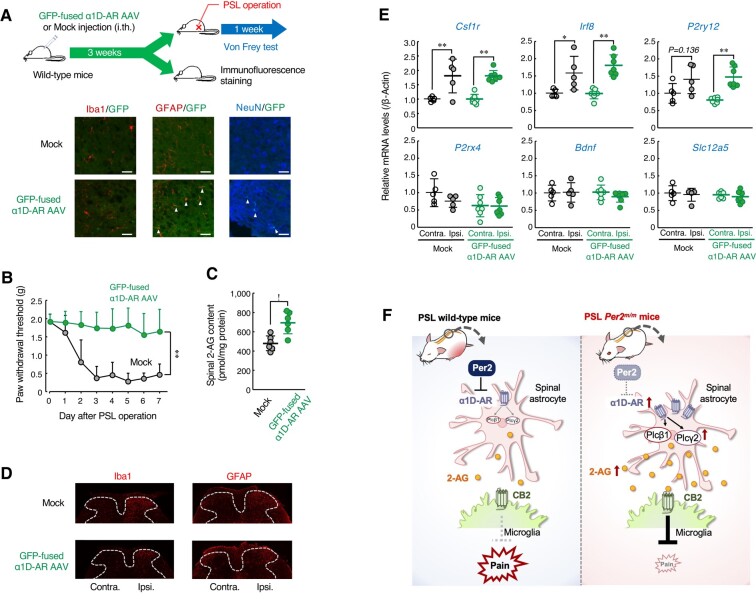
Spinal expression of α1D-AR alleviates the PSL-induced tactile pain hypersensitivity in wild-type mice. A) The upper panel indicates the experimental protocol. The below panel shows the localization of GFP-fused α1D-AR. Double immunofluorescence labeling of GFP-fused α1D-AR with Iba1, a marker of microglia; GFAP, a marker of astrocytes; and NeuN, a marker of neurons in the spinal slices of male wild-type mice at 3 weeks after intrathecal injection of GFP-fused α1D-AR AAV. GFP-tagged α1D-AR positive cells were double labeled with GFAP and NeuN are indicated by white arrows. Scale bar, 10 µm. B) The withdrawal threshold of ipsilateral hind paws of male PSL-wild-type mice injected with GFP-fused α1D-AR AAV. The PWT was assessed at ZT10. Values are shown as means with SD (*n* = 6–13). **; *P* < 0.01, significant difference between the two groups (group; *F*_1,136_ = 171.667; *P* < 0.001, day; *F*_7,136_ = 11.811; *P* < 0.001, interaction; *F*_7,136_ = 6.665; *P* < 0.001, two-way ANOVA with Tukey–Kramer's post hoc test). C) Spinal 2-AG content in male PSL-wild-type mice injected with GFP-fused α1D-AR AAV. The 2-AG content was assessed at ZT10. Values are shown with SD (*n* = 6). *P < 0.05, significant difference between the two groups (Student's t test). D) Microglia and astrocyte states in the contralateral (contra.) and ipsilateral (ispi.) spinal dorsal horn of PSL-wild-type male mice injected with GFP-fused α1D-AR AAV. Immunofluorescent staining with Iba1 or GFAP in the spinal cord at ZT10. The dorsal horn areas are surrounded by the dashed line. Scale bar, 10 µm. E) The mRNA levels of *Csf1r*, *Irf8*, *P2ry12*, *P2rx4*, *Bdnf*, and *Slc12a5* in the contralateral (contra.) and ipsilateral (ispi.) spinal cord of male PSL-wild-type mice injected with GFP-fused α1D-AR AAV. The mRNA levels were assessed at ZT10. Values are shown with SD (*n* = 5–7). **P < 0.01, *P < 0.05, significant difference between the two groups (*F*_3,20_ = 14.422, *P* < 0.001 for *Csf1r*, *F*_3,20_ = 12.730, *P* < 0.001 for *Irf8*, *F*_3,20_ = 2.072, *P* = 0.1136 for *P2rx4*, *F*_3,20_ = 8.503, *P* < 0.001 for *P2ry12*, *F*_3,20_ = 0.547, *P* = 0.656 for *Bdnf*, *F*_3,20_ = 0.427, *P* = 0.736 for *Slc12a5*; one-way ANOVA with Tukey–Kramer's post hoc test). F) Schematic illustrating the underlying mechanism for the failure to PSL-induced tactile pain hypersensitivity in male *Per2^m/m^* mice. Up-regulation of spinal α1D-AR expression in *Per2^m/m^* mice increases 2-AG production through activation of Plcβ1 and Plcγ2. The 2-AG acts on the cannabinoid receptor 2 (CB2), which suppresses the PSL-induced tactile pain hypersensitivity.

In the final set of experiments, we investigated whether increased expression of α1D-AR attenuates tactile allodynia of wild-type mice following PSL. To achieve this, male wild-type mice were injected intrathecally with adeno-associated viral (AAV) vectors expressing GFP-fused mouse α1D-AR. Although our constructed AAV vectors expressed transgenes under the control of the cytomegalovirus (CMV) promoter, immunofluorescence of GFP-fused α1D-AR was double labeled with GFAP and NeuN, but not with Iba1 (Fig. 5A). This was probably due to the inability of AAV vectors to express transgenes in microglia (34). Spinal expression of α1D-AR prevented the PSL-induced tactile allodynia in wild-type mice (Fig. 5B), accompanied by enhancement of 2-AG production in the spinal cord (Fig. 5C), but the α1D-AR expression had a negligible effect on acute pain behavior of male wild-type mice (Fig. S1C). Moreover, α1D-AR-expressing PSL-wild-type mice showed similar morphological or molecular alterations in spinal glial cells to those observed in control vector (mock)-injected mice (Fig. 5D and E). These results suggest that enhancement of α1D-AR-mediated 2-AG production in the spinal cord prevents the PSL-induced tactile pain hypersensitivity in male wild-type mice. Therefore, increased spinal expression of α1D-AR in *Per2^m/m^* mice may contribute to their insensitivity to neuropathic pain.

## Discussion

In general, disruption of the circadian rhythm has been implicated in the onset or exacerbation of various diseases (35). Neuropathic pain has also been thought to be exacerbated by disruption of the circadian clock; however, our present results demonstrated that a deficiency in the *Per2* gene prevents the PSL-induced tactile pain hypersensitivity. The increased expression of α1D-AR in the spinal cord of *Per2^m/m^* male mice promotes the production of 2-AG, which activates the cannabinoid receptor and prevents neuropathic tactile allodynia (Fig. 5F).

The activation and increase in the number of glial cells in the dorsal horn of the spinal cord is one of the characteristics of the development of neuropathic tactile allodynia. Although the activation state of microglia in the rat brain has been shown diurnal variation owing to stimuli by noradrenaline and glucocorticoids (36), our previous study demonstrated no significant time-dependent variation in the number of microglia and astrocytes in the spinal cord of PSL-wild-type male mice (27). As observed in male wild-type mice, male *Per2^m/m^* mice also exhibited the activation of glial cells in the dorsal horn of the spinal cord after PSL, indicating that the peripheral nerve injury-induced activation pathways of spinal glial cells are not affected by the deficiency of *Per2* gene. On the other hand, the *Per2* gene is involved in the regulation of sleep and the development of depression (37). Thus, the altered neuronal activity associated with pain transmission may also occur in *Per2^m/m^* mice, which could suppress the production of pain hypersensitivity. However, intrathecal injection of α1D-AR antagonist or PLC inhibitor produced pain hypersensitivity in PSL-*Per2^m/m^* mice. The facts support the notion that the pain transmission pathway of *Per2^m/m^* mice functions similarly to that of wild-type animals.

The descending noradrenergic pathway is a route that transmits signals from the locus coeruleus to the spinal cord and plays an important role in the chronic pain state (38). The spinal expression of the α1D-AR was increased in *Per2^m/m^* mice, while noradrenaline levels in the spinal cord of *Per2^m/m^* mice were similar to those of wild-type mice. A previous report has demonstrated that the expression level of α1D-AR is lower than that of other α1 AR subtypes (39). Despite the lower expression level, the affinity of α1D-AD for noradrenaline is ∼20 times higher than that of other α1 receptor subtypes (40). The expression level of α1D-AR in the spinal cord of *Per2^m/m^* mice was ∼1.5-hold higher than that of wild-type mice, but administration of an α1D-AR antagonist to PSL-*Per2^m/m^* mice resulted in producing tactile allodynia associated with a reduction of 2-AG levels. This suggests that even slight changes in the expression level of α1D-AR can cause significant alterations in signal transduction because of the high affinity of this receptor for noradrenaline.

Per2 cooperates with other clock gene products to control the circadian rhythm of various gene expressions. Mice with dysfunctional clock genes show not only altered rhythms of various gene expressions but also the changes in the basal expression levels for genes that do not exhibit circadian rhythms (41, 42). Although the increase in the expression of α1D-AR in *Per2^m/m^* mice was observed only in the spinal cord and kidneys, the expression did not show a significant diurnal rhythm in the spinal cord of wild-type mice (Fig. S4). In cultured astrocytes, the expression levels of α1D-AR were decreased by infections with a retrovirus expressing the *Per2* gene, indicating that Per2 acts as negative regulator for α1D-AR expression. Therefore, Per2 may cooperate with a certain factor expressed in spinal cells and kidneys to repress α1D-AR expression.

The functional role of α1D-AR in the central nervous system is not fully understood yet. In this study, we identified two enzymes, Plcβ1 and Plcγ2, involved in the biosynthesis of 2-AG that serve as downstream factors of the α1D-AR. The α1D-AR belongs to the GPCR family that couples with Gq protein to activate PLC (31). Indeed, it has been suggested that stimulation of α1 receptors in cardiac myocytes not only activates PLC but also increases its expression level (43). Although the enzymatic activity of Plcβ1 and Plcγ2 in the spinal cord could not be assessed owing to the small tissue size, intrathecal injection of an α1D-AR antagonist led to a decrease in the mRNA levels of these 2-AG synthases as well as a suppression of 2-AG production. Therefore, stimulation of α1D-AR in the spinal cord may lead to an increase in 2-AG production by regulating the expression of Plcβ1 and Plcγ2.

2-AG exerts physiological effects through cannabinoid receptors CB1 and CB2 (44). Intrathecal injection of CB2 antagonist into PSL-*Per2^m/m^* mice produced tactile allodynia. CB2 is mainly expressed in microglia of the central nervous system (45, 46) and plays a critical role in the suppression of neuropathic pain (47). Therefore, CB2-mediated signaling in the microglial cells of male *Per2^m/m^* mice is functioning normally to alleviate neuropathic tactile allodynia. On the other hand, a previous study suggests that CB2 is involved in the activation pathway of cultured microglia prepared from cerebral cortices of Sprague–Dawley pups (48). Recently, several subtypes of microglia have been identified with different functions, including in a mechanism for the remission or recurrence of neuropathic pain (49, 50). As observed in wild-type mice, sciatic nerve ligation in *Per2^m/m^* mice induced the expression of genes related to neuropathic pain development and activation of microglia, but CB2 may be primarily expressed in the spinal microglia that regulates the remission of neuropathic pain. Further studies are needed to determine which subtypes of spinal microglia express CB2.

The endocannabinoids are known to be involved in the analgesic system that also relieves nociceptive pain (51, 52). Despite an increase in 2-AG levels, male *Per2^m/m^* mice showed similar responses to wild-type mice in the hot plate and capsaicin tests (Fig. S1A). The intrathecal injection of 2-AG (50 µg/mouse) into PSL-wild-type mice prevented tactile allodynia, but the same amount of 2-AG injection was ineffective against heat- and capsaicin-induced pain in wild-type mice (Fig. S1B). Furthermore, α1D-AR overexpression in the spinal cord of wild-type mice also had a negligible effect on acute pain (Fig. S1C). Administration of higher doses of 2-AG may suppress heat- and capsaicin-induced pain in wild-type mice, but the increase in 2-AG levels by AAV-induced expression of α1D-AR in the spinal cord of wild-type mice did not appear to suppress of acute pain. Similarly, activation of the endocannabinoid system by increasing 2-AG in the spinal cord of *Per2^m/m^* mice may also be insufficient for suppressing acute pain. On the other hand, arachidonic acid produced after degradation of 2-AG has been suggested to exacerbate neuropathic pain (53). The spinal expression of Mgl was increased in *Per2^m/m^* mice, but they failed to exhibit tactile pain hypersensitivity even after injury of the sciatic nerve. Furthermore, the results of mass spectrometry analysis also showed no significant accumulation of arachidonic acid in the spinal cord of *Per2^m/m^* mice (Table S1). These observations suggest that the increased spinal expression of Mgl mRNA in *Per2^m/m^* mice is not essential for the enhancement of arachidonic acid production.

In addition to the male *Per2^m/m^* mice in the institute of cancer research (ICR) background and male wild-type ICR mice were used in this study, we also had attempted to investigate the development of tactile pain hypersensitivity in *Per2^m/m^* female mice following PSL. However, the mean value of PWT of PSL-*Per2^m/m^* female mice exhibited a large individual variation that may hinder an accurate evaluation of the role of the *Per2* gene in the pathological states of neuropathic pain. Different mechanisms have been proposed for the development of neuropathic tactile allodynia in male and female mice (54). Spinal microglia plays a critical role in the development of nerve injury-induced tactile allodynia in male mice, whereas peripheral T lymphocytes make a significant contribution to the production of mechanical pain hypersensitivity in female mice. Dysfunction of the *Per2* gene also affects immune function (55–57) and may induce large individual variations in the contribution of T lymphocytes to the nerve injury-induced pain hypersensitivity.

The expression of α1D-AR was identified in the human spinal cord (58). Considering the basal mechanism of the circadian clock is also conserved across species, the suppression of neuropathic pain development induced by circadian clock dysfunction in animal models is likely to occur in humans. Understanding this mechanism is critical for appropriate patient care and the identification of effective analgesic targets. Our findings reveal an unappreciated influence of circadian machinery dysfunction on neuropathic pain and provide effective therapeutic strategy for the treatment of chronic pain disorders.

## Materials and methods

### Animals and treatments

Male *Per2^m/m^* mice in the ICR background and male wild-type ICR mice (5–10 weeks old) were housed in groups (from 5 to 7 per cage) under light (200 Lux) and dark (<1 Lux) cycles (lights on from ZT0 to ZT12) at 24 ± 1 °C and 60 ± 10% humidity with food and water ad libitum. During the dark period, a dim red light (<10 Lux) was used to aid animal treatment. Under inhalational isoflurane anesthesia (concentration: 3–5%), animals underwent PSL as follows (26). The right thigh was shaved, and the sciatic nerve was exposed through an incision. Half of the nerve was tightly ligated with 7-0 silk thread and the wound was sutured (ipsilateral side; right hind paw). The left hind paw was nontreated (contralateral side; left hind paw). The surgery was performed from ZT6 to ZT10. All animal experiments were conducted in accordance with the Guidelines for Animal Experiments of Kyushu University and approved by the Institutional Animal Care and Use Committee of Kyushu University (approved protocol ID #A22-019).

### Cell culture and treatment

Murine neuroblastoma C1300, murine microglial cell line MG-6, and murine immortalized astrocytes were maintained in Dulbecco’s modified Eagle medium (Life Technologies Inc., Carlsbad, CA, USA) supplemented with 5% fetal bovine serum (FBS) (Biowest, Nuaillé, France) and 0.25% penicillin–streptomycin (FUJIFILM Wako Pure Chemical, Tokyo, Japan) at 37 °C in a humidified 5% CO_2_ atmosphere. Astrocytes were infected with retroviral particles expressing *Per2* (kindly provided by Dr Nakao, Yamanashi University). To select clones stably expressing *Per2*, cells were maintained in a medium containing 2 μg/mL of puromycin. PER2 expression was confirmed by western blotting.

### Assessment of mechanical allodynia

To assess mechanical allodynia, mice were placed individually in an opaque plastic cylinder, which was placed on a wire mesh and habituated for 0.5 h to allow acclimatization to the new environment. Calibrated von Frey filaments (0.02–2.0 g, North Coast Medical) were then applied to the plantar surfaces of the hind paws of mice. The PWT was evaluated using the up-down method (26). Observers were blinded to genetic background and drug treatment. To examine the diurnal variation of pain hypersensitivity, the PWT of mice was assessed at ZT2, ZT6, ZT10, ZT14, ZT18, and ZT22. Before assessment, mice were habituated for 0.5 h, and they were returned to their cages after assessment. This procedure was repeated every 4 h.

### Drug administration

Intraperitoneal (i.p.) injection was performed by administering the drug into the peritoneal cavity of the mice using a syringe with a 30-G needle. DSP-4 (50 mg/kg; Cayman Chemical, Ann Arbor, MI, USA) was dissolved in phosphate-buffered saline (PBS) containing 2% ethanol and administered intraperitoneally (59, 60). The intrathecal injection was performed using a Hamilton syringe attached to a 30-G needle, and the 10 μL of the drug was injected between the L4 and L5 lumbar segments of the spinal cord over 30 s. The following drugs were injected intrathecally at ZT10, during which neuropathic allodynia was exacerbated in laboratory rodents (27). The dosage of each drug was determined based on previous reports: prazosin (30 nmol in 10 µL sterile purified water; Cayman Chemical) (61); yohimbine (10 nmol in 10 μL saline; FUJIFILM Wako Pure Chemical) (62); propranolol (100 nmol in 10 μL saline; FUJIFILM Wako Pure Chemical) (63); WB4101 (100 nmol in 1% dimethyl sulfoxide; Cayman Chemical) (64); L-765,314 (0.25 nmol in saline; MedChemExpress, Monmouth Junction, NJ, USA); BMY7378 (1 fmol in 10 μL saline; Abcam, Cambridge, UK); AM281 (0.2 nmol in 10 µL in 1% dimethyl sulfoxide; R&D Systems, Minneapolis, MN, USA); JTE907 (0.2 nmol in 10 µL in 1% dimethyl sulfoxide; Santa Cruz Biotechnology, Santa Cruz, CA, USA); and U73122 (10 nmol in 1% dimethyl sulfoxide; Abcam) (65).

### Quantitative RT-PCR analysis

RNA was extracted from mouse lumbar spine segment from L4 to L5 at ZT10 and cultured astrocytes using RNAiso reagent (Takara Bio Inc., Osaka, Japan). cDNA was synthesized using a ReverTra Ace qPCR RT kit (Toyobo Life Science, Osaka, Japan). For quantitative real-time RT-PCR, cDNA was amplified by PCR using the LightCycler 96 system (Roche Diagnostics, Mannheim, Germany) and THUNDERBIRD NEXT SYBR qPCR Mix (Toyobo Life Science). PCR primer sequences are listed in Table S2.

### Western blotting

Total protein was prepared from the L4/L5 lumbar spine segment of mice at ZT10 and cultured astrocytes using CelLytic MT (Sigma-Aldrich) according to the manufacturer's instructions. Denatured samples containing 10–30 μg of protein were separated by sodium dodecyl sulfate-polyacrylamide gel electrophoresis (SDS–PAGE) and then transferred onto poly vinylidene fluoride (PVDF) membranes. Membranes were reacted with primary antibodies against α1D-AR (1:1,000; sc-390884; Santa Cruz Biotechnology), PER2 (1:3,000; 313132A3; Alpha Diagnostic International, San Antonio, TX, USA), and β-ACTIN (1:10,000; sc-1616-HRP; Santa Cruz Biotechnology). Specific antigen–antibody complexes were visualized using horseradish peroxidase-conjugated secondary antibodies and ImmunoStar reagent (FUJIFILM Wako Pure Chemical). The visualized images were scanned using an ImageQuant LAS4010 (GE Healthcare, Tokyo, Japan).

### Immunohistochemical staining

Mice were deeply anesthetized by inhalation of isoflurane at a concentration of 3–5%. After the disappearance of the flip-tail reflex, the abdomen was immediately incised and 30 mL of cold PBS was perfused through the heart, followed by 30 mL of cold 4% paraformaldehyde solution in PBS. These treatments were conducted from ZT10 to ZT12. The tissues were fixed on ice for 1 h, and then the spinal lumbar segments from L4 to L5 were removed and further fixed with 4% paraformaldehyde solution at 4 °C for 12 h. The fixed tissues were then dehydrated by incubation in 15 and 30% sucrose at 4 °C for 12 h. After immersion in 30% sucrose, dehydrated tissues were embedded in O.T.C. compound (Sakura Finetek, Torrance, CA, USA) and stored at −80 °C until for experiments. Coronal 14 μm thick sections of the spinal cord were prepared using a frozen sectioning device (Thermo Scientific-Cryostar NX70, Reinach, Switzerland). The sections were then washed and blocked with PBS containing 5% goat serum (Cedarlane, Burlington, Canada), 1.25 M glycine (FUJIFILM Wako Pure Chemical), and 0.1% Triton X-100. The samples were incubated with primary antibodies against Iba1 (1:1,000; 01919741; FUJIFILM Wako Pure Chemical), GFAP (1:1,000; 13-0300; Thermo Fisher Scientific, Waltham, MA, USA), or NeuN (1:1,000; MAB377; Sigma-Aldrich) for 48 h at 4 °C. After incubation with primary antibodies, the samples were washed with PBS containing 0.1% Triton X-100, and secondary antibody reactions were performed with fluorescently labeled antibodies against rat IgG (1:2,000; A-11081; Alexa Fluor 546, Thermo Fisher Scientific or ab150154; Alexa Flour 555, Abcam), rabbit IgG (1:2,000; A11008; Alexa Fluor 488, Thermo Fisher Scientific or ab150078; Alexa Flour 555, Abcam) or mouse IgG (1:2,000; ab150107; Alexa Flour 647, Abcam) at room temperature for 2 h. After incubation, sections were washed with 0.1% Triton X-100. The sections were mounted with Vectashield (Vector Laboratories, Burlingame, CA, USA). Fluorescence images were obtained using confocal fluorescence microscopy, and the number of Iba1-positive microglial cells and GFAP-positive astrocytic cells was quantified with BZ-X800 Analyzer cell counter analysis tool in defined area of interest on the spinal cord dorsal. Scoring was blinded to animal treatment.

### Dissociation of spinal cord and cell sorting

Under isoflurane inhalation anesthesia, the spinal cord was removed from mice as described above and treated with HBSS (+) containing 10% FBS, 0.2 U/mL collagenase D (Roche Diagnostics Burgess Hill, UK), and 2.4 U/mL dispase (Thermo Fisher Scientific) for 30 min at 37 °C. Spinal tissue samples were homogenized by passing through a 23-G needle attached to a 1-mL syringe and further incubated at 37 °C for 15 min. After incubation, the samples were homogenized by passing twice through a 26-G needle attached to a 1-mL syringe, and the enzymatic reaction was stopped by adding 0.5 M ethylenediaminetetraacetic acid (EDTA). To prepare microglia and astrocytes, myelin debris was removed from the cell suspension using Myelin Removal Beads II and a MACS LS column (Miltenyi Biotec, Bergisch-Gladbach, Germany), according to the manufacturer's protocol. Samples were centrifuged (400 × *g*, 5 min, 4 °C) and resuspended with ice-HBSS(-) containing 2% FBS. The cell suspension was blocked by adding TruStain FcX PLUS (BioLegend, San Diego, CA, USA) and incubating for 10 min at 4 °C. For sorting microglia and astrocytes, the cell suspension was blocked by incubation with Fc Block and stained with PE antimouse/human CD11b (BioLegend, RRID:AB_312791), PerCP antimouse CD45 (BioLegend, RRID:AB_312977), APC antimouse ACSA-2 (Miltenyi Biotec, 130-116-245), and FITC antimouse Ly6G (BioLegend, RRID:AB_1236494) for 1 h at 4 °C in the dark. After washing, samples were acquired using a BD FACSAria III Cell Sorter (Becton Dickinson Biosciences, San Diego, CA, USA) with FACSDiva software v6.1.3. We used a Neuron Isolation Kit and a MACS LS column (Miltenyi Biotec) to obtain neurons according to the manufacturer's protocol.

The separation of each cell fraction was confirmed by the detection of mRNA for marker genes. Total RNA was extracted from the neuron fraction using the RNAiso Plus (Takara Bio Inc) and from microglia and astrocytes using ReliaPrep RNA Cell Miniprep System (Promega, Madison, WI, USA) according to the manufacturer's instructions. cDNA was synthesized using a ReverTra Ace qPCR RT kit (Toyobo Life Science). The cDNA encoding CD11b, a marker of microglia, Sox9, a marker of astrocytes, and NeuN, a marker of neurons, were amplified using GoTaq Green Master Mix (Promega) with the gene-specific primers listed in Table S3. The PCR-amplified products were separated by electrophoresis using 2% agarose gel containing ethidium bromide. Signals from the agarose were detected by exposure to UV light.

### Determination of noradrenalin in the spinal cord

Under isoflurane inhalation anesthesia, the spinal cords were collected from mice at ZT10 and then homogenized with 100 µL of 0.1 M HClO_4_. The lysates were centrifuged at 12,000 *×g* for 15 min, and the supernatants were used as the sample for LC/MS/MS analysis. NE was resolved using a Hypersil GOLD PFP column (150 mm × 2.1 mm, 50 mm, part no. 25402-152130, Thermo Fisher Scientific) and an AQUITY UPLC H-Class XHCLQT0100 system (Waters), consisting of a vacuum degasser, binary pump, thermostatically controlled column compartment, thermostatically controlled autosampler, and a diode array detector. Mobile phase A consisted of 0.1% formic acid/water; mobile phase B consisted of 0.1% formic acid/acetonitrile. A linear gradient was generated at 0.5 mL/min: 0.0 min, 0% A and 100% B, 2.0 min, 5% A, and 95% B; 3.0 min, 5% A, and 95% B; 7.0 min, 100% A, and 0% B (Waters application note). The injection volume was 10 μL. The column temperature was controlled at 35 °C and the autosampler compartment was set to 4 °C. An AQUITY TQ mass spectrometer controlled by MassLynx software (Waters) was operated in a selected reaction monitoring mode. The multiple reaction monitor was set at a mass-to-charge ratio (*m*/*z*) of 152–107 *m*/*z* (cone voltage: 50 V, collision voltage: 20 V).

### Mass spectrometry analysis for lipid mediators

Under isoflurane inhalation anesthesia, the spinal cords were collected from mice at ZT10, and samples were homogenized in 200 µL ice-cooled deionized water. The homogenate (150 µL) was mixed with 600 µL ethyl acetate/*n*-hexane (9:1, v/v), and vortexed. After centrifugation at 14,000*×g* for 5 min (4°C), the upper layer was collected and evaporated using a centrifugal evaporator. Samples were dissolved into 50 µL-aliquots of the mobile phase and analyzed using LC-MS/MS systems, Agilent 6495C triple quadrupole LC/MS (Agilent Technologies, Santa Clara, CA, USA). Chromatographic separations were performed under gradient conditions at a flow rate of 0.2 mL/min using a Luna Omega PS C_18_ column (SHIMADZU, Kyoto, Japan). The mobile phase was water and acetonitrile (40:60 v/v) containing 0.1% formic acid. Quantitation was performed by multiple reaction monitoring (MRM) in negative ion mode. The levels of each substance were expressed as ratio to the internal standard per milligram of total protein measured using the Pierce BCA Assay Kit (Thermo Fisher Scientific).

### Production of α1D-AR-expressing AAV vectors

Recombinant AAV vectors expressing the mouse α1D-AR gene fused with eGFP under the control of CMV promoter were produced from HEK293 cells by triple transfection with the Rep/Cap plasmid, the pAAV2/9 transgene plasmid, and the adenoviral helper plasmid. The vectors were purified by two cesium chloride density gradient purification steps and dialyzed against PBS containing 0.001% (v/v) Pluronic-F68. A total of 5.5 × 10^9^ genomic copies of AAV vectors were injected intrathecally into the L4/L5 lumbar spine segments of mice. Three weeks after the injection of the AAV vectors, the spinal cords of the mice were collected to evaluate the expression of GFP-fused α1D-AR. Mice that received a total of 2.75 × 10^9^ genomic copies of AAV vector injection underwent PSL and were used for behavioral tests, immunohistochemical staining, and qRT-PCR.

### Statistical analysis

Results are expressed as mean with SD. The significance of differences among groups was analyzed by one- or two-way ANOVA followed by Tukey, Tukey–Kramer's, or Dunnett's post hoc test. The two-sided unpaired Student's t test was used for independent comparison between the two groups. Statistical analysis was performed using JMP Pro 14.0.0 (SAS Institute Inc., Cary, NC, USA). A probability level of *P* < 0.05 was considered to be significant. To present representative findings are presented, similar results were obtained at least three times.

## Supplementary Material

pgad482_Supplementary_Data

## Data Availability

The data supporting the findings of this work are available within the article and its supplementary material.
