# Supplementary material for: Suppression of neuropathic pain in the circadian clock–deficient Per2m/m mice involves up-regulation of endocannabinoid system
Source: PNAS Nexus. 2024 Jan 17;3(1):pgad482. doi: 10.1093/pnasnexus/pgad482 (PMC10794166; doi:10.1093/pnasnexus/pgad482)
Supplement: pgad482_Supplementary_Data [file pgad482_supplementary_data.zip › PNASNEXUS-PNASNEXUS-2023-00908R-s01.docx]

**Supporting Information for**

**Suppression of neuropathic pain in the circadian clock deficient Per2m/m mice involves upregulation of endocannabinoid system**

Wakaba Yamakawa, Sai Yasukochi, Yuya Tsurudome, Naoki Kusunose, Yuta Yamaguchi,

Akito Tsuruta, Naoya Matsunaga, Kentaro Ushijima, Satoru Koyanagi and Shigehiro Ohdo

This file includes text for Supporting methods and Supplementary Figures S1 to S6

**Supplementary methods**

**Acute pain test.** Mice were placed individually in a plastic cage and habituated for 1 h to allow acclimatization to the environment. After adaptation, a hot plate test and capsaicin test were performed from ZT6 to ZT10. 2-AG [924894-97-3, Cayman chemical, 50 µg in 10 µL dimethyl sulfoxide/olive oil (1:19)] was intrathecally injected into mice 1 h before behavior tests. α1D-AR-expressing vector (5.5 × 10^9^ genomic copies) was intrathecally injected into mice three weeks before behavior tests. A hot plate test was performed on the plate at 49°C or 52°C, and the times of the initiation reactions, such as paw licking, biting, and jumping were recorded. The cutoff time was 1 min. For the capsaicin test, capsaicin (1 μg in 20 μL PBS, FUJIFILM Wako Pure Chemical) was injected into the right hind paw, and the length of time spent licking, biting, and shaking hindpaw was measured for 5 min.

**Determination of serotonin in the spinal cord.** Serotonin was measured according to the determination of noradrenaline. The multiple reaction monitor was set at a mass-to-charge ratio (m/z) of 177–115 m/z (cone voltage:15V, collision voltage:25V) for 5-HT.


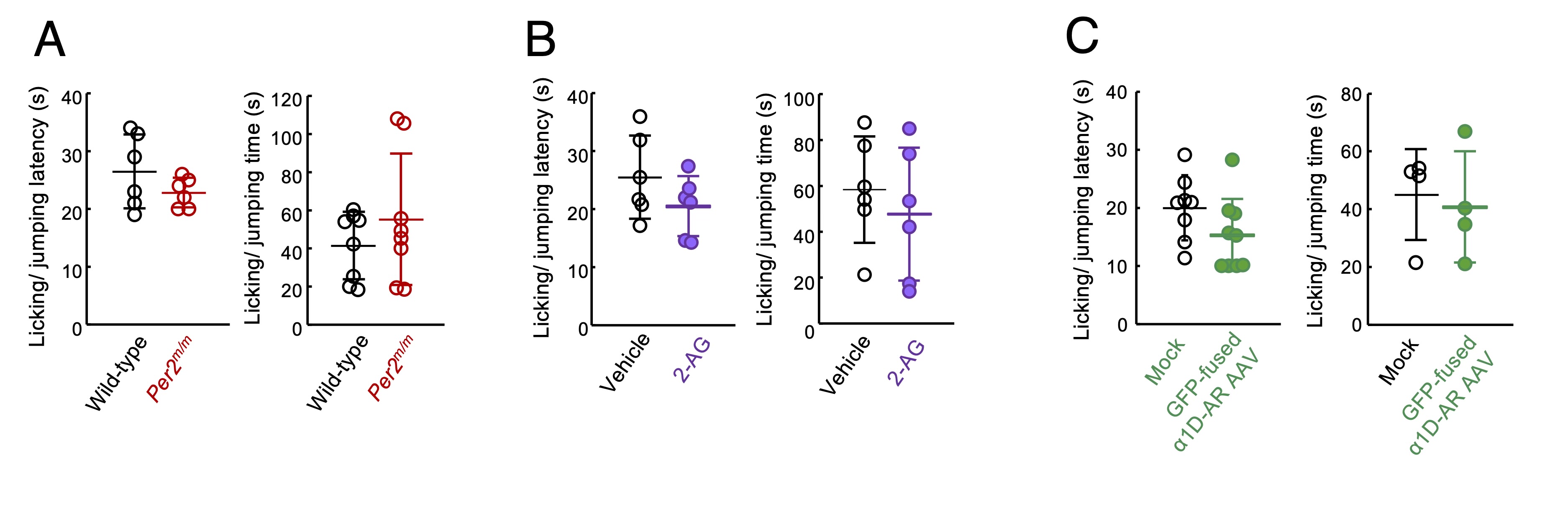


**Supplementary Figure S1 No significant modulation of acute pain behaviors by Per2 gene mutation, 2-AG administration, and spinal expression of α1D-AR.** (**A**) Acute pain behaviors of male wild-type and male *Per2^m/m^* mice were assessed by hot plate test (left) and capsaicin test (right) from ZT10 to ZT12. Values are shown as means with S.D. (n = 6-8). (**B**) Acute pain behaviors of male wild-type mice after intrathecal injection of 2-AG (50 µg) 1 h before behavior tests. Pain behaviors were assessed by hot plate test (left) and capsaicin test (right) at 1 h after 2-AG injection. Values are shown as means with S.D. (n=6). (**C**) Acute pain behaviors of α1D-AR expressing male wild-type mice. Pain behaviors were assessed by hot plate test (left) and capsaicin test (right) at 3 weeks after intrathecal injection of GFP-fused α1D-AR AAV. Values are shown as means with S.D. (n = 4-9).


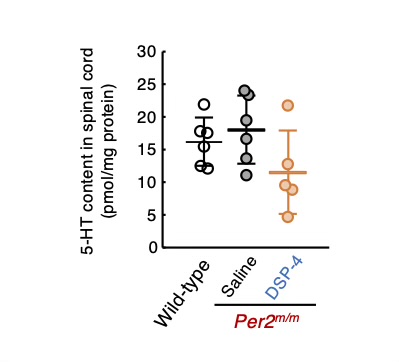


**Supplementary Figure S2 Spinal serotonin (5-HT) content of male wild-type and DSP-4 administered male *Per2*^m/m^ mice.** The 5-HT content was assessed at ZT10 on day 7 after DSP-4 administration. Values are shown as mean with S.D. (n = 5-6).

**
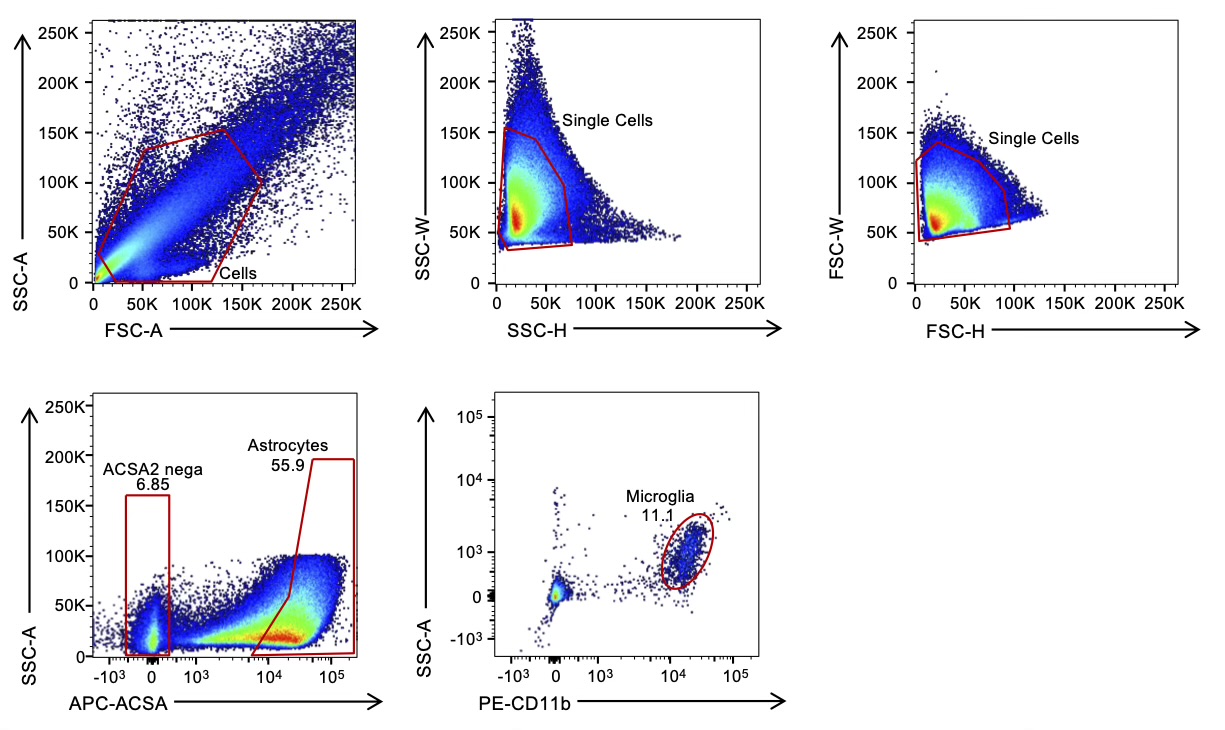
**

**Supplementary Figure S3 Identify 2-AG producing cells in the spinal cord.** Gating strategy for sorting of astrocytes and microglia from the spinal cord.

**
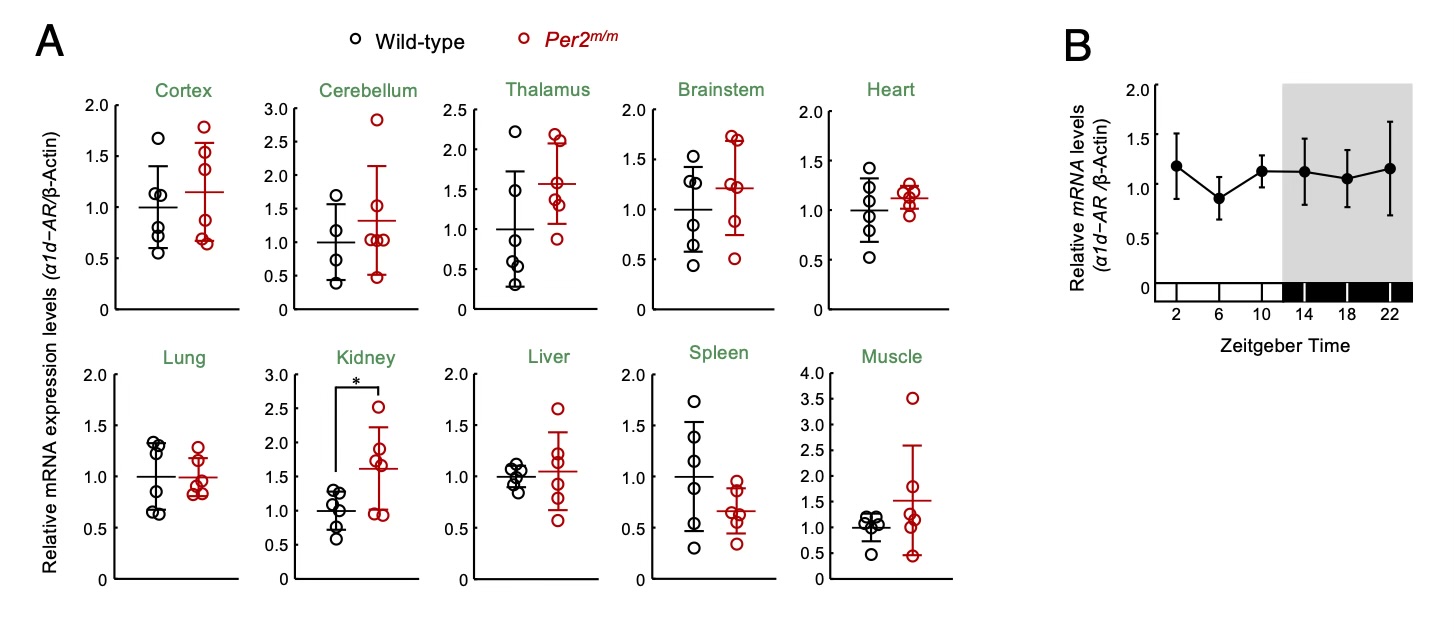
**

**Supplementary Figure S4 The mRNA expression profile of *α1D-AR* in wild-type and *Per2*^m/m^ mice.** (**A**) The mRNA levels of α1D-AR in various organs of male wild-type and male *Per2^m/m^* mice at ZT10. Values are shown with S.D. (n = 4-6). *; *P* < 0.05, a significant difference between the two groups (Student’s *t*-test). (**B**) Temporal profiles of the *α1D-AR* mRNA in the spinal cord of male wild-type mice. Values are shown as mean with S.D. (n = 6). There was no significant time-dependent variation (*F*_5,28_ = 0.882; *P* = 0.506, one-way ANOVA).


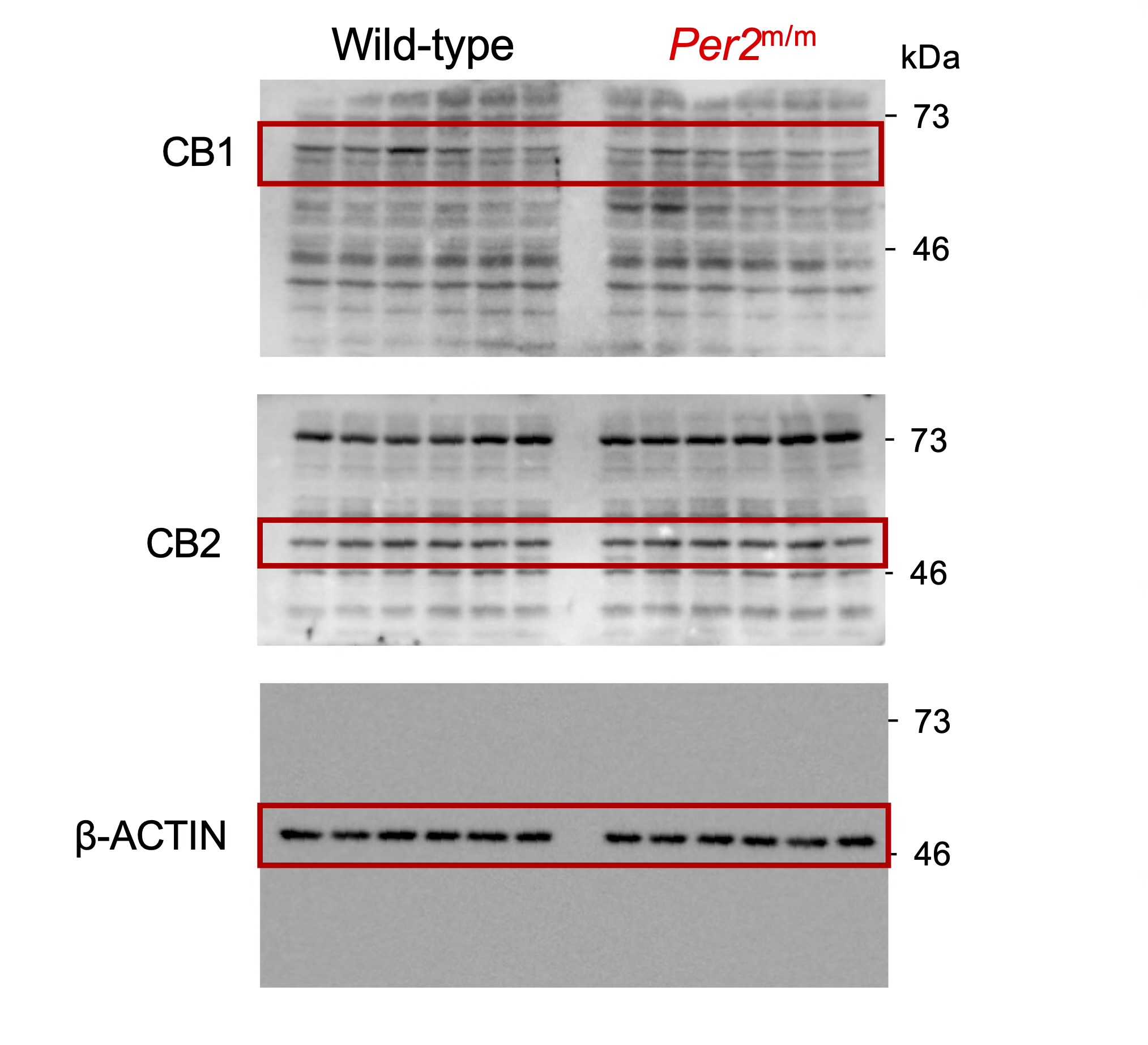
**Supplementary Figure S5** Unedited full blots of Figure 3B

**Supplementary Figure S6** Unedited full blots of Figure 4D
