# Supplementary material for: Suppression of neuropathic pain in the circadian clock–deficient Per2m/m mice involves up-regulation of endocannabinoid system
Source: PNAS Nexus. 2024 Jan 17;3(1):pgad482. doi: 10.1093/pnasnexus/pgad482 (PMC10794166; doi:10.1093/pnasnexus/pgad482)
Supplement: pgad482_Supplementary_Data [file pgad482_supplementary_data.zip › PNASNEXUS-PNASNEXUS-2023-00908R-s02.pdf]

Supplementary Table S1 Mass spectrometry analysis for lipid mediators in the spinal cord of wild-type and *Per2*$^{m/m}$ mice after injection of α1D-AR antagonist BMY7378 or saline.

Name	Transition (m/z)	Wild-type (Saline)	*Per2*$^{m/m}$ (Saline)	*Per2*$^{m/m}$ (BMY7378)	Statistical significance
11,12-EET-EA	346 → 62	0.19 ± 0.04	0.12 ± 0.06	0.15 ± 0.05	N.S.
11-HETE	319 → 167	0.9 ± 0.51	0.85 ± 0.37	0.87 ± 0.39	N.S.
12,13-DiHOME	313 → 183	6.71 ±2.53	5.69 ± 0.76	9.67 ± 3.44	N.S.
12-HHT	279.2 → 179.1	2.89 ± 0.92	2.56 ± 0.86	3.92 ± 1.29	N.S.
13-HODE	295.2 → 195.1	4.02 ± 1.04	3.65 ± 1.44	5.96 ± 1.88	N.S.
15-HETE	319 → 219	1.48 ± 0.89	0.87 ± 0.21	1.36 ± 0.49	N.S.
2-AG	287.2 → 379.4	233.24 ± 80.30	386.19 ± 111.58*	182.17 ± 84.64$^{\#\#}$	*P<0.05, $^{\#\#}P$<0.01
6-keto-PGF1α	369 → 163	33.82 ± 6.65	29.50 ± 5.90	33.68 ± 7.67	N.S.
8,15-DiHETE	335.2 → 127.1	2.46 ± 0.73	1.95 ± 0.96	3.12 ± 0.65	N.S.
8-iso-PGE2	351.2 → 271.5	4.76 ±1.50	4.79 ± 0.98	7.32 ± 2.38	N.S.
9,10-DiHOME	313 → 201	9.58 ± 3.60	7.05 ± 1.21	12.95 ± 3.27$^{\#}$	$^{\#}P$<0.05
9-HODE	295 → 183	1.29 ± 0.42	1.07 ± 0.41	1.35 ± 0.23	N.S.
AA	303 → 205	1.43 ± 1.34	0.94 ± 0.48	1.37 ± 0.42	N.S.
AEA	348.3 → 62	10.33 ± 3.82	11.56 ± 2.13	10.30 ± 3.13	N.S.
DHA	327.2 → 229.2	0.87 ± 0.29	1.13 ± 0.36	1.73 ± 0.46$^{\#\#}$	$^{\#\#}P$<0.01
dOEA	328.7 → 62.2	2.43 ± 0.30	2.76 ± 0.38	2.70 ± 0.27	N.S.
EPA	301.2 → 257.2	1.91 ± 1.04	0.98 ± 0.29	1.58 ± 0.34	N.S.
LBT4-EA	362.3 → 189.1	0.76 ± 0.15	0.65 ± 0.29	0.95 ± 0.24	N.S.
OEA	326.3 → 62.1	595.97 ± 151.21	751.96 ± 142.35	628.14 ± 180.25	N.S.
PGD2	351.2 → 271.5	4.18 ± 1.62	4.61 ± 0.81	6.30 ± 1.56	N.S.
PGE1	353.2 → 235.2	4.11 ± 0.78	3.48 ± 1.12	6.68 ± 1.47$^{\#\#}$	$^{\#\#}P$<0.01
PGE2	351.2 → 271.5	3.98 ± 1.31	4.47 ± 1.09	5.50 ± 1.35	N.S.
PGF2α	353 → 193	1.79 ± 1.36	0.87 ± 0.21	1.33 ± 0.41	N.S.
TXB2	369 → 169	1.12 ± 0.31	1.04 ± 0.27	1.65 ± 0.45$^{\#}$	$^{\#}P$<0.05

The levels of each substance were expressed as ratio to the internal standard per mg of total protein. Values represent the mean ± S.D. (n=5-6). $*P<0.05$, significant difference from saline-injected wild-type mice. $^{\#\#}P<0.01$, significant difference from saline-injected *Per2*$^{m/m}$ mice. (ANOVA with Tukey-Kramer’s post hoc test).

Abbreviations: 11,12-EET-EA; 11(12)-EET ethanolamide: 11-HETE; hydroxyeicosatetraenoic acids:12,13-DiHOME; 12,13-dihydroxy-9Z-octadecenoic acid: 12-HHT;12-hydroxy-heptadecatrienoic acid: 13-HODE; 13-hydroxyoctadecadienoic acid: 15-HETE; 15-hydroxyeicosatetraenoic acids: 2-AG; 2-arachidonoylglycerol: 6-keto-PGF1α; 6-keto prostaglandin F1α: 8,15-DiHETE; 8,15-dihydroxyeicosatetraenoic acids: 8-iso-PGE2; 8-iso-prostaglandin E2: 9,10-DiHOME; 9,10-dihydroxy-9Z-octadecenoic acid: 9-HODE;9-hydroxyoctadecadienoic acid: AA; arachidonic acid: AEA; anandamide: DHA; docosahexaenoic acid: EPA; eicosapentaenoic acid:LBT4-EA; leukotriene B4 ethanolamide: OEA; N-oleoylethanolamine: PGD2; prostaglandin D2: PGE1; prostaglandin E1: PGE2; prostaglandin E2: PGF2α; prostaglandin F2α: TXB2; thromboxane B2;

Supplementary Table S2 Primer sequences for RT-PCR analysis

Gene symbol	Primer
Mouse *β-Actin*	Forward: 5'- ACTGTCGAGTCGCGTCC -3'<br>Reverse: 5'- CGCAGCGATATCGTCATCCAT-3'
Mouse *Irf8*	Forward: 5'- GGATATGCCGCCTATGACACA -3'<br>Reverse: 5'- CATCCGGCCCATACAACTTAG -3'
Mouse *P2ry12*	Forward: 5'- CACAGAGGGCTTTGGGAACTTA -3'<br>Reverse: 5'- TGGTCCTGCTTCTGCTGAATC -3'
Mouse *P2rx4*	Forward: 5'- ACAACGTGTCTCCTGGCTACAAT -3'<br>Reverse: 5'- GTCAAACTTGCCAGCCTTTCC -3'
Mouse *Bdnf*	Forward: 5'- GCCCAACGAAGAAAACCATAAG -3'<br>Reverse: 5'- TGTTTGCGGCATCCAGGTA -3'
Mouse *Csf1r*	Forward: 5'- TGTCATCGAGCCTAGTGGC -3'<br>Reverse: 5'- GGTCCAAGGTCCAGTAGGG -3'
Mouse *Slc12a5*	Forward: 5'- GGGCAGAGAGTACGATGGC -3'<br>Reverse: 5'- CCCTGGGGTAGGTTGGTGTA -3'
Mouse *Cnr1*	Forward: 5'- AAGTCGATCTTAGACGGCCTT -3'<br>Reverse: 5'- TCCTAATTTGGATGCCATGTCTC -3'
Mouse *Cnr2*	Forward: 5'- ATGGCCGTGCTCTATATTATCCT -3'<br>Reverse: 5'- ATGGTCACACTGCCGATCTTC -3'
Mouse *Mgll*	Forward: 5'- CGGACTTCCAAGTTTTTGTCAGA -3'<br>Reverse: 5'- GCAGCCACTAGGATGGAGATG -3'
Mouse *Dagla*	Forward: 5'- GTCCTGCCAGCTATCTTCCTC -3'<br>Reverse: 5'- CGTGTGGGTTATAGACCAAGC -3'
Mouse *Dagb*	Forward: 5'- AGCGACGACTTGGTGTTCC -3'<br>Reverse: 5'- GCGTGAGATACAACGTCAGACT -3'
Mouse *Plcb1*	Forward: 5'- AGACCTGGTGAACATTTCCCA -3'<br>Reverse: 5'- ACAAGCCTCTAGTGCAGTTTC -3'
Mouse *Plcb2*	Forward: 5'- TGCTGATCGAAAACGGGTGG -3'<br>Reverse: 5'- AGCTTTAGAGTGGTAGGAAGTGA -3'
Mouse *Plcb3*	Forward: 5'- CGCGGGAGTAAGTTCATCAAA -3'<br>Reverse: 5'- AGAAGCCATTAGGGTCCACAC -3'
Mouse *Plcb4*	Forward: 5'- AGTGCTAGAATGTTCCCTCATCA -3'<br>Reverse: 5'- GAAGCCGATATTCACCAGATCC -3'
Mouse *Plcg1*	Forward: 5'- GAGACGCGCCAGATCACAT -3'<br>Reverse: 5'- AAAGTCCCGAGAAGTCTTCCC-3'
Mouse *Plcg2*	Forward: 5'- GTGGACACCCTTCCAGAATATG -3'<br>Reverse: 5'- GGGCGTTGAACACGGTCAT -3'
Mouse *Adra1a*	Forward: 5'- CGGTGACTCACTACTACATTGTC -3'<br>Reverse: 5'- GACGCTGTGCAGCATAAGAC -3'
Mouse *Adra1b*	Forward: 5'- ACATTGGGGTGCGATACTCTC -3'<br>Reverse: 5'- TTGGGCGCAGGTTCTTTCC -3'
Mouse *Adra1d*	Forward: 5'- CGCTGTGGTGGGAACCGGCAG -3'<br>Reverse: 5'- AGTTGGTGACCGTCTGCAAGT -3'
Mouse *Adra2a*	Forward: 5'- GGTGACACTGACGCTGGTTT -3'<br>Reverse: 5'- ACTGGTGAACACCGCGATAATA -3'
Mouse *Adra2b*	Forward: 5'- TCTTCACCATTTTCGGCAATGC -3'<br>Reverse: 5'- AGAGTAGCCACTAGGATGTCG -3'
Mouse *Adra2c*	Forward: 5'- CCTACTGGTACTTCGGGCAAG -3'<br>Reverse: 5'- CGGTCCAGACTAATGGCACA -3'

Mouse *Adrb1*	Forward: 5'- CAGAAGGCGCTCAAGACACT -3'<br>Reverse: 5'- GAAGACGAAGAGGCGATCCG -3'
Mouse *Adrb2*	Forward: 5'- AATAGCAACGGCAGAACGGA -3'<br>Reverse: 5'- GTCAACGCTAAGGCTAGGCA -3'
Mouse *Adrb3*	Forward: 5'- CTCCTCGTAATGCCACCAGG -3'<br>Reverse: 5'- CGCACAAGGTCTCGATGCTA -3'

Supplementary Table S3 Sequences of primer set for identification cell fraction

Gene symbol	Primer
Mouse *18S*	Forward: 5'- CGGCTACCACATCCAAGGAA -3'<br>Reverse: 5'- GCTGGAATTACCGCGGCT -3'
Mouse *Rbfox*	Forward: 5'- GGCAAATGTTCGGGCAATTCG -3'<br>Reverse: 5'- TCAATTTTCCGTCCCTCTACGAT -3'
Mouse *CD11b*	Forward: 5'- AGTGCTGGGAGACGTGAATG -3'<br>Reverse: 5'- GCACTGAGGCTGGCTATTGA -3'
Mouse *Sox9*	Forward: 5'- CAGCCCCTTCAACCTTCCTC -3'<br>Reverse: 5'- TGATGGTCAGCGTAGTCGTATT -3'
Mouse *Adra1d*	Forward: 5'- CGGACCTTCTGCGACGTATG -3'<br>Reverse: 5'- TGGCTGGATACTTGAGCGAGT -3'
